# Activation of cannabinoid receptor type 2-induced osteogenic differentiation involves autophagy induction and p62-mediated Nrf2 deactivation

**DOI:** 10.1186/s12964-020-0512-6

**Published:** 2020-01-15

**Authors:** Aihua Xu, Yang Yang, Yang Shao, Meng Wu, Yongxin Sun

**Affiliations:** Department of Rehabilitation Medicine, The First Affiliated Hospital of China Medical University, 155 North Nanjing Street, Shenyang, Liaoning 110001 People’s Republic of China

**Keywords:** Osteoporosis, Osteoblast, CNR2, Autophagy, p62, Nrf2 signaling pathway

## Abstract

**Background:**

Dysfunction in survival and differentiation of osteoblasts commonly occurs in patients with osteoporosis. Cannabinoid receptor type 2 (CNR2) is a major receptor of endocannabinoid system that is crucial for bone mass homeostasis. Our group prior demonstrated that activation of CNR2 signaling promoted osteogenic differentiation of bone marrow derived mesenchymal stem cells in vitro. Autophagy is reported to participate in osteoblastic differentiation. Whether autophagy is regulated by CNR2-mediated cannabinoid signaling is unknown, and how the autophagy-CNR2 interaction affects osteoblastic differentiation requires further elucidation.

**Methods:**

hFOB 1.19 osteoblasts were treated with CNR2 agonists HU308 (5, 10, 25, 50 or 100 nM) and JWH133 (1, 2, 5, 10 or 20 μM) in presence or absence of autophagy inhibitor 3-Methyladenine (3-MA). The differentiation of hFOB 1.19 cells was determined via evaluating their alkaline phosphatase (ALP) activity and mineralization ability (Alizarin red staining). Alterations in autophagy-related molecules and osteogenic markers were analyzed via real-time PCR and/or immunoblotting assays.

**Results:**

hFOB 1.19 cells spontaneously differentiated towards mature osteoblasts under 39 °C, during which CNR2 expression increased, and autophagy was activated. The strongest autophagy flux was observed at 192 h post differentiation─LC3I to LC3II conversion was enhanced and Beclin 1 expression was upregulated considerably, while p62 expression was downregulated. Treatment of HU308 and JWH133 promoted autophagy in a dose-dependent manner, and suppressed mTOR signaling pathway in hFOB 1.19 cells. In CNR2-silenced cells, HU308’s and JWH133’s effects on autophagy were weakened. HU308 and JWH133 enhanced the ALP activity and mineralization, and upregulated the expression of osteogenic markers, osteopontin and osteocalcin, in hFOB 1.19 cells. Intriguingly, such pro-osteogenic effects induced by CNR2 activation were markedly mitigated by 3-MA. In addition to provoking autophagy, CNR2 agonists also reduced nuclear Nrf2 accumulation and increased Keap1 expression. Further, re-expression of p62 inhibited CNR2 agonists-induced Nrf2 degradation.

**Conclusions:**

Osteogenic differentiation induced by CNR2 signaling activation involves autophagy induction and p62-mediated Nrf2 deactivation.

## Background

Osteoporosis is a systemic skeletal disorder featured by structural deterioration of bone mass and reduction of bone mineral density, and it is a silent disease until a fracture occurs [1, 2]. Excessive use of glucocorticoids [3] and deficiency of estrogen [4] are two major clinical risk factors that augment the risk of fragility fracture in aged population and postmenopausal women, respectively. Dysfunction in survival and differentiation of osteoblasts, a cell population responsible for generating extracellular matrix proteins and regulating matrix mineralization, is commonly found in patients with osteoporosis [5]. Hence, osteoblasts are considered as a target of anti-osteoporotic therapy [6].

Cannabinoid receptor type 2 (CNR2) is a G protein-coupled receptor that binds to endogenous cannabinoids [7]. By giving osteoporotic animals with synthetic CNR2 agonists or antagonists, several previous studies revealed a conflicting role of CNR2-mediated cannabinoid signaling in regulating the homeostasis of bone mass. Idris and co-workers proved that AM630, a CNR2-selective antagonist, mitigated ovariectomy-induced bone loss in mice [8]. This team further demonstrated that mice lacking CNR1 and CNR2 had higher trabecular bone mass and displayed resistance to ovariectomy-induced osteoporosis [9]. They deduced that the negative regulatory role of activated CNR2 signaling in bone formation was associated with enhanced osteoclast formation. Ofek and colleagues, on the contrary, demonstrated that CNR2-deficient mice had significant age-related trabecular bone loss [10]. They also found that HU308, a CNR2-selective agonist, mitigated ovariectomy-induced bone loss [10]. Our prior work showing that activation of CNR2 signaling induced by UR144 augmented the osteogenic differentiation of bone marrow-derived mesenchymal stem cells (BMMSCs) [11] supported an osteogenesis-promoting role of CNR2. We here further elucidated the underlying mechanisms with a focus on autophagy and Nuclear Factor Erythroid 2 Related Factor 2 (Nrf2) signaling pathway.

Cells often initiate autophagy to deliver damaged organelles or protein aggregates to vacuole or lysosome where the cargos are degraded and recycled [12]. Recently, activation of autophagy is reported to enhance the osteogenic differentiation. The mineralization of BMMSCs was suppressed when autophagy was inhibited, whereas enhanced when autophagy was re-activated [13]. How CNR2 signaling transduction affects autophagy during osteogenic differentiation is unclear. Nevertheless, several previous lines of evidence imply that CNR2 agonists can regulate autophagy flux. In macrophages and cardiomyocytes, HU308 was found to enhance cellular autophagy [14, 15]. We therefore proposed that activation of CNR2 signaling may enhance the osteogenic differentiation by provoking autophagy.

Under quiescent condition, Kelch-like ECH-associated protein 1 (Keap1), binds to Nrf2, a transcription factor, and induces its constitutive degradation through the ubiquitin-proteasome pathway [16, 17]. Keap1-deficient mice exhibited hyperactivation of Nrf2 and had impaired bone formation [18]. Such negative regulatory role of over-activated Nrf2 is suggested to link to its inhibitory effects on osteoblast differentiation [19]. The Sequestosome 1 (SQSTM1)/p62 (hereafter referred to p62) is a common component of protein aggregates, and it can be recruited to ubiquitinated protein aggregates [20, 21]. During dynamic autophagy, p62 functions as a bridge between autophagosomes and polyubiquitinated cargo, and p62 itself eventually becomes degraded [22]. Interestingly, p62 contains a Keap1-interacting region (KIR) similar to Nrf2, which enables p62 to tone up Nrf2 signaling transduction through competitively binding to Keap1 [23]. We here also investigate whether CNR2 agonists affect Nrf2 signaling transduction, and whether p62 is involved in such process.

Herein, hFOB 1.19 osteoblasts were treated with two synthetic CNR2 ligands, HU308 and JWH133, and their differentiation was determined by analyzing the cell mineralization, activity of alkaline phosphatase (ALP) and expression of bone formation markers. We found that osteogenic differentiation induced by CNR2 activation was inhibited when autophagy was blocked with 3-MA. CNR2 agonists reduced Nrf2 expression in hFOB 1.19 cells by accelerating p62 degradation.

## Materials and methods

### Cell culture and RNA interference (RNAi) of CNR2

Human osteoblastic hFOB 1.19 cells (Procell, Wuhan, China) were routinely maintained in DMEM/F12 medium (Procell) containing 10% fetal bovine serum (FBS; Sigma-Aldrich, St. Louis MO, USA) and 0.3 mg/ml G418 under 5% CO_2_ and 95% air at 34 °C. hFOB 1.19 cells were cultured at 34 °C until reaching confluence, and then transferred to 39 °C as described previously [24]. After being cultured at 39 °C for 48 h, cells were further treated with HU308 (5, 10, 25, 50 or 100 nM; purity ≥98% determined with HPLC; Tocris Bioscience, Minneapolis, MN, USA) or JWH133 (1, 2, 5, 10 or 20 μM; purity ≥98% determined with HPLC; Tocris Bioscience) for additional 12 h, 96 h or 192 h. As 100 nM HU308 and 20 μM JWH133 contained 0.05% DMSO, to eliminate the effects induced by the solvent, all cell culture media contained 0.05% DMSO.

CNR2 short hairpin RNA (CNR2 shRNA-S, 5′ ttcatcaactccatggtcaattcaagagattgaccatggagttgatgattttttc 3′; CNR2 shRNA-AS, 5′ tcgagaaaaaatcatcaactccatggtcaatctcttgaattgaccatggagttgatgaa 3′) was inserted into pSico lentiviral shuttle plasmid (Addgene,Cambridge, MA, USA) between HpaI and XhoI sites. Then pSico-CNR2 shRNA plasmid, psPAX2 package plasmid and pMD2.G envelope plasmid were co-transfected into 293 T cells to generate CNR2 shRNA lentiviruses (LV-CNR2 shRNA). hFOB 1.19 cells were infected with LV-CNR2 shRNA at 34 °C, and 24 h later, cells were transferred to 39 °C for differentiation. Forty-eight hours later, cells were further treated with 50 nM HU308 or 10 μM JWH133 for 12 h, and then harvested.

Human BMMSCs obtained from ZqxzBio (Shanghai, China) were cultured in MSC specific culture medium according to the supplier’s protocols. Osteogenic differentiation of BMMSCs was induced according to our prior work [11]. BMMSCs were cultured in osteoinductive media with or without 50 nM HU308 and 10 μM JWH133. BMMSCs were infected with LV-CNR2 shRNA or control lentiviruses, and 24 h later, they were incubated in osteoinductive media for 48 h. Then, BMMSCs were stimulated with 50 nM HU308 and 10 μM JWH133 for 12 h, and then collected.

### Alkaline phosphatase (ALP) activity

ALP activity was assessed via a commercial kit (Nanjing Jiancheng Bioengineering Institute, Jiangsu, China) according to manufacturer’s protocols.

### Western blotting analysis

Total proteins were isolated from the whole lysates of cells with cell lysis buffer **(**Beyotime, Shanghai, China), and from nuclear and cytoplasmic fractions with the Nuclear and Cytoplasmic Protein Extraction Kit (Beyotime, Shanghai, China). The concentrations were analyzed with a BCA Protein Assay Kit (Beyotime). Protein complex was separated by SDS-PAGE, and transferred onto PVDF (Millipore, Bedford, MA, USA) membranes. After blocking with skim milk (5% M/V), the membranes were incubated with one of the primary antibodies at 4 °C overnight: anti-CNR2 (1:500; Abclonal, Shanghai, China), anti-Secreted Phosphoprotein 1 (SSP1)/osteopontin (1:500; Proteintech, Wuhan, China), anti-bone gamma-carboxyglutamate (gla) protein (BGLAP)/osteocalcin (1:1000; Abcam, Cambridge, MA, USA), anti-Nrf-2 antibody (1:500; Proteintech), anti-Lamin B (1:3000; Proteintech), anti-LC3II/I antibody (1:1000; Cell Signaling Technology, Danvers, MA, USA); anti-Beclin 1 (1:1000; Proteintech), anti-p62 antibody (1:2000; Proteintech), anti-Keap 1 (1:3000; Proteintech), anti-phosphorylated Mammalian Target Of Rapamycin (p-mTOR; 1:500, CST, Danvers, MA, USA), anti-mTOR (1:1000, CST), anti- phosphorylated Ribosomal Protein S6 Kinase 70 kDa Polypeptide 1 (p-P70S6K; 1:500, CST), anti-p70S6K antibody (1:1000, CST), anti-phosphorylated Eukaryotic Translation Initiation Factor 4E Binding Protein 1 (p-4EBP1; 1:500, Abclonal) and anti-4EBP1 (1:1000; Affinity, Cincinnati, OH, USA) and GAPDH (1:10000; Proteintech). Then, the PVDF membranes were incubated with horseradish peroxidase-conjugated goat anti-rabbit secondary antibody (1: 5000; Beyotime) for 45 min at 37 °C. To the end, protein blots were visualized with enhanced chemiluminescence (ECL; Beyotime), and their intensities were analyzed with a gel imaging system. Relative protein expression levels were normalized to GAPDH or Lamin B.

### Reverse transcription (RT)-PCR and real-time RT-PCR

Total RNAs were isolated with TriPure isolation reagent (Bioteke, Beijing, China), and processed into cDNAs via Super M-MLV reverse transcriptase (Bioteke). PCR products were analyzed in agarose gel (1.5%) electrophoresis. The relative gene expression levels were determined by real-time RT-PCR. In short, cDNA templates were mixed with primers (Genscript, Nanjing, China), Sybr Green (Sigma-Aldrich, St. Louis, MO, USA) and 2 × Power Taq PCR MasterMix (Bioteke), and analyzed on an Exicycler™ 96 PCR instrument. Housekeeping gene GAPDH was used as a reference. Primer information was as following: CNR2–F 5′–agccctcatacctgttcattgg–3′; CNR2–R 5′–gtgaaggtcatagtcacgctg–3′; osteopontin–F, 5′–gaagtttcgcagacctgacat–3′, osteopontin–R 5′–gtatgcaccattcaactcctcg–3; osteocalcin–F, 5′ –cactcctcgccctattggc–3′, osteocalcin–R, 5′–ccctcctgcttggacacaaag–3′. Data from real-time PCR results were calculated via the 2^−ΔΔCt^ method.

### Immunofluorescent staining

Cell dishes were washed with 1× PBS (Sangon, Shanghai, China) for several times, and fixed in 4% paraformaldehyde (Sinopharm, Shanghai, China) for 15 min at room temperature. Then, cells were permeabilized with 0.1% Triton X-100 (Beyotime) at room temperature for 30 min, and blocked with goat serum for 15 min. Thereafter, cell dishes were incubated with anti-Nrf-2 antibody (1:100) or anti-p62 antibody (1:100) overnight at 4 °C. Afterwards, these cells were incubated with Cy3-labled secondary antibody (1:200; Beyotime) for 45 min at 37 °C. The fluorescent pictures were taken with an Olympus camera (DP73).

### Alizarin red staining

Mineralization of hFOB 1.19 cells were detected with Alizarin red staining. In short, cells seeded in 96-well plates were incubated with 50 nM HU308, 10 μM JWH133 or 2 mM 3-MA (MCE, Monmouth Junction, NJ, USA) for 8 d, and fixed in 4% paraformaldehyde and dyed by Alizarin red S (Solarbio, Beijing, China).

### CCK-8 assay

To determine whether 2 mM 3-MA treatment affected cell vitality, CCK-8 assay was performed in hFOB 1.19 cells cultured at 39 °C for 96 h or 192 h. In brief, cells were treated with CCK-8 reagent (Beyotime) for 1 h, and then the absorption values at 450 nm were analyzed with a microplate reader.

### Statistical analysis

Values in each histogram were presented as mean ± standard deviation. Statistical significance was determined with one-way analysis of variance followed by Tukey’s analysis (GraphPad Prism 7.0; GraphPad software, La Jolla, CA, USA). Data in Fig. 3 were analyzed with two-way analysis of variance followed by Sidak’s analysis (GraphPad Prism 8.0). Symbols ^*,**,***^ indicated a *p* value < 0.05, 0.01, and < 0.001.

## Results

### Expression alterations in CNR2, autophagy molecules, and Nrf2 during osteogenic differentiation in vitro

**Fig. 1:**
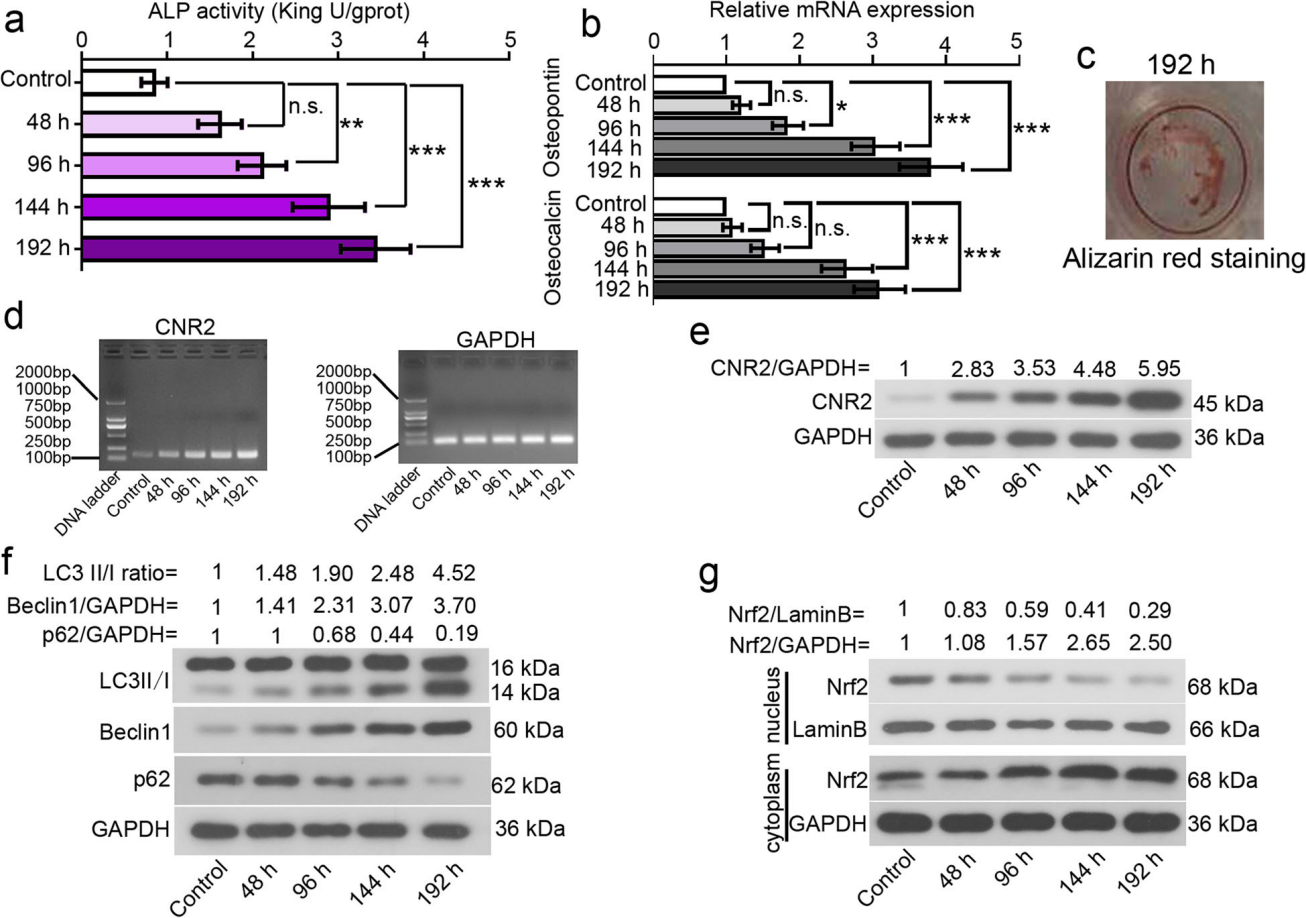
Alterations in CNR2, autophagy molecules, and Nrf2 during osteogenic differentiation in vitro*.* To stimulate osteogenic differentiation, hFOB 1.19 cells were transferred from 34 °C to 39 °C, and cultured for indicated periods. **a** ALP activities of hFOB 1.19 cells were determined via a commercial kit. **b** The expression levels of osteopontin and osteocalcin, two osteogenic markers, were analyzed with real-time PCR. **c** Cell mineralization was determined with Alizarin red staining. **d** mRNA expression levels of CNR2 and GAPDH were determined with RT-PCR. **e**-**g** Protein levels of CNR2, LC3, beclin 1, p62 and Nrf2 (nuclear and cytoplasmic) were determined with western blotting, and normalized to that of GAPDH or laminB. Symbols*, ** and *** indicated a *p* value < 0.05, < 0.01 and < 0.001

ALP activities were determined in hFOB 1.19 cells incubated at 39 °C for 48, 96, 144 or 192 h. As seen in Fig. 1a, ALP activity increased with time. The expression levels of osteopontin and osteocalcin, two osteogenic markers, were analyzed with real-time PCR. Upregulation in these two molecules was observed in differentiated hFOB 1.19 cells (Fig. 1b). Alizarin red staining showed that a 192-h osteoinductive differentiation promoted obvious cell mineralization (Fig. 1c). These data together confirmed that hFOB 1.19 cells had osteogenic differentiation potential at 39 °C.

CNR2 expression was determined with RT-PCR and western blotting. The corresponding data showed that CNR2 mRNA and protein expression levels increased during osteogenic differentiation (Fig. 1d-e). Further, we noted effective autophagy flux in differentiated hFOB 1.19 cells─LC3II/I conversion was enhanced and beclin 1 expression was upregulated, whilst p62 expression was downregulated (Fig. 1f). In addition, less nuclear and more cytoplasmic Nrf2 were detected in differentiated hFOB 1.19 cells (Fig. 1g). These data reveal activation of CNR2 and autophagy signaling, and deactivation of Nrf2 signaling during the spontaneous osteogenic differentiation.

### CNR2 agonists enhance osteogenic differentiation by activating autophagy

**Fig. 2:**
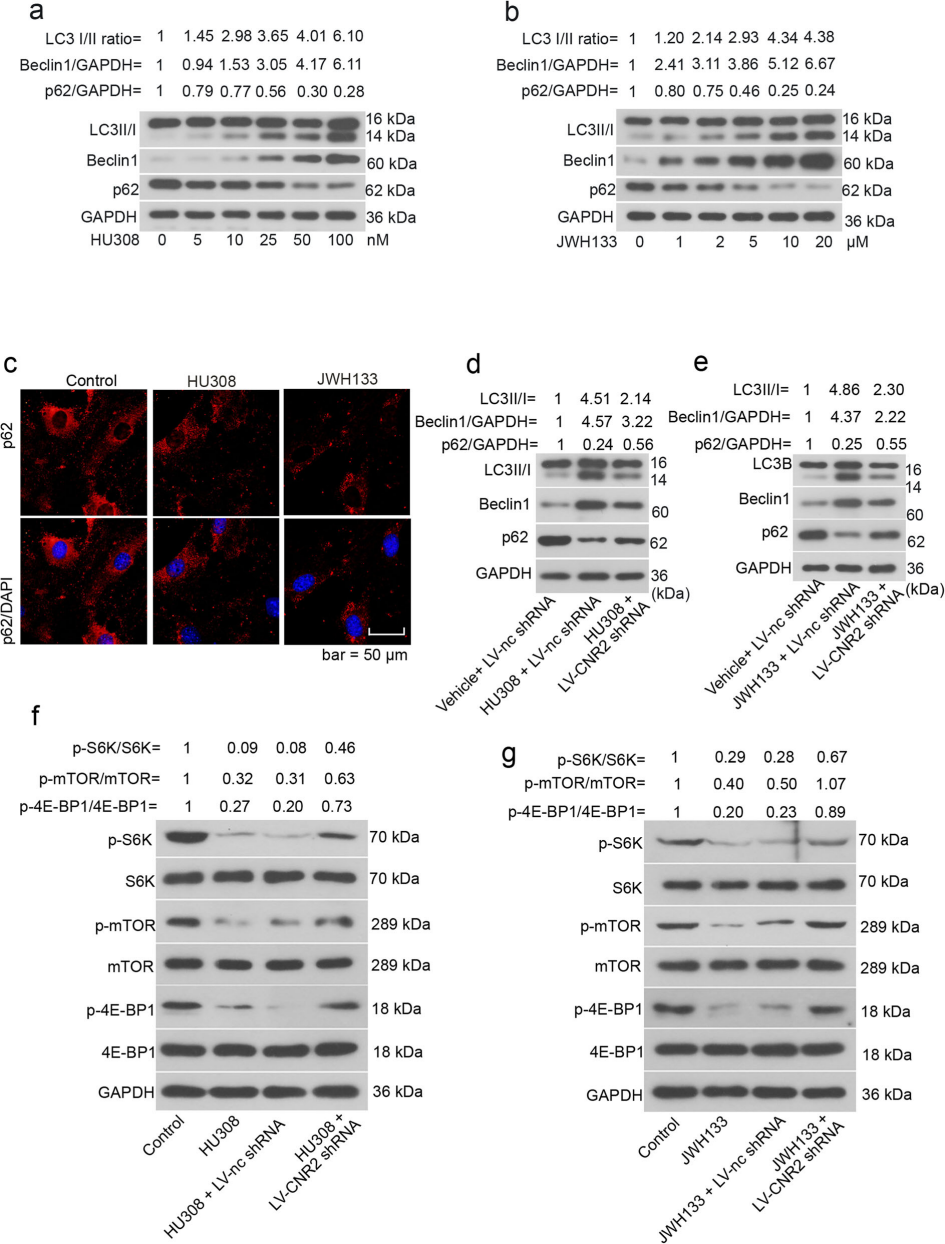
CNR2 agonists activate autophagy and suppress mTOR pathway in hFOB 1.19 cells. hFOB 1.19 cells were cultured at 34 °C until reaching confluence, and then transferred to 39 °C. Forty-eight hours later, HU308 (5, 10, 25, 50 or 100 nM) or JWH133 (1, 2, 5, 10 or 20 μM) was added into the cell medium. Twelve hours later, hFOB 1.19 cells were subjected to **a**-**b** western blotting analysis. **c** For immunofluorescence staining of p62, hFOB 1.19 cells were treated with 50 nM HU308 or 10 μM JWH133 for 12 h. **d**-**g** hFOB 1.19 cells were infected with LV-CNR2 shRNA at 34 °C, and 24 h later, cells were transferred to 39 °C for differentiation. Forty-eight hours later, cells were further treated with 50 nM HU308 or 10 μM JWH133 for 12 h, and then harvested for western blotting analysis

hFOB 1.19 cells were cultured at 39 °C for 48 h, and then treated with two CNR2 agonists, HU308 and JWH133, for additional 12 h. Data from western blotting indicated that the CNR2 agonists promoted the conversion of LC3I to LC3II, upregulated beclin 1 expression, and accelerated p62 degradation in hFOB 1.19 cells (Fig. 2a-b). Immunofluorescence images validated the p62 degradation in hFOB 1.19 cells induced by HU308 and JWH133 (Fig. 2c). CNR2 agonists-promoted autophagy was weaken by CNR2 knockdown (Fig. 2d-e). Further, CNR2 agonists suppressed mTOR signaling pathway as evidenced by decreased phosphorylation of mTOR, P70S6K and 4EBP1 in hFOB 1.19 cells (Fig. 2f-g). The signaling transduction of mTOR pathway was partly restored when CNR2 was silenced (Fig. 2f-g). Similar alterations were also observed in human BMMSCs (Additional file 1: Figure S1a-b).

**Fig. 3:**
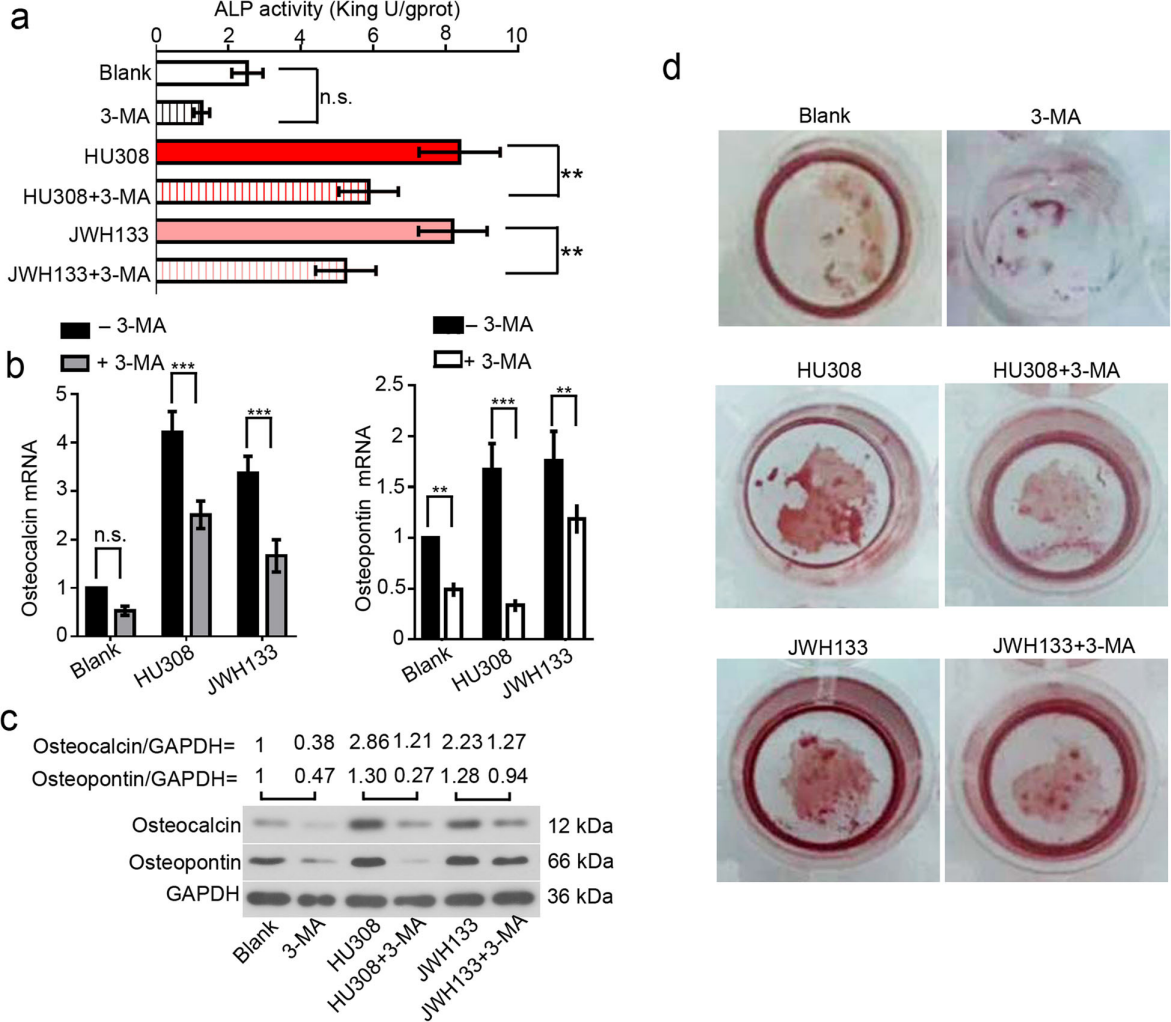
CNR2 agonists-induced osteogenic differentiation is blocked by autophagy inhibitor 3-MA. hFOB 1.19 cells were cultured at 34 °C until reaching confluence, and transferred to 39 °C. These cells were then treated with 2 mM 3-MA, 50 nM HU308 or 10 μM JWH133 for **a**-**c** 96 h or **d** 192 h. **a** The ALP activity was determined, and shown as mean ± standard deviation. The mRNA and protein levels of osteocalcin and osteopontin were determined with **b** real-time RT PCR and **c** western blotting, respectively. Data were shown as mean ± standard deviation. Cell mineralization was determined with Alizarin red staining. Heavier staining indicated stronger mineralization

We next blocked cellular autophagy with 3-MA, and evaluated the effects of HU308 and JWH133 on hFOB 1.19 cell osteogenic differentiation. The treatment of 2 mM 3-MA hardly affected cell viability (Additional file 2: Figure S2). ALP activity significantly increased in cells exposed to HU308 or JWH133, and decreased in response to 3-MA (Fig. 3a). Further, the expression levels of two osteogenic differentiation markers, osteocalcin and osteopontin, were upregulated by CNR2 agonists, and downregulated by 3-MA (Fig. 3b-c). hFOB 1.19 cell mineralization determined with Alizarin red staining was enhanced by CNR2 agonists, and weakened by 3-MA (Fig. 3d). Likewise, 3-MA inhibited CNR2 agonists-induced osteogenic differentiation of BMMSCs (Additional file 1: Figure S1c-e).

These data together demonstrate that CNR2 agonists-induced osteogenic differentiation is attenuated when autophagy is hindered.

### CNR2 agonists inhibit nuclear Nrf2 expression during osteogenic differentiation

**Fig. 4:**
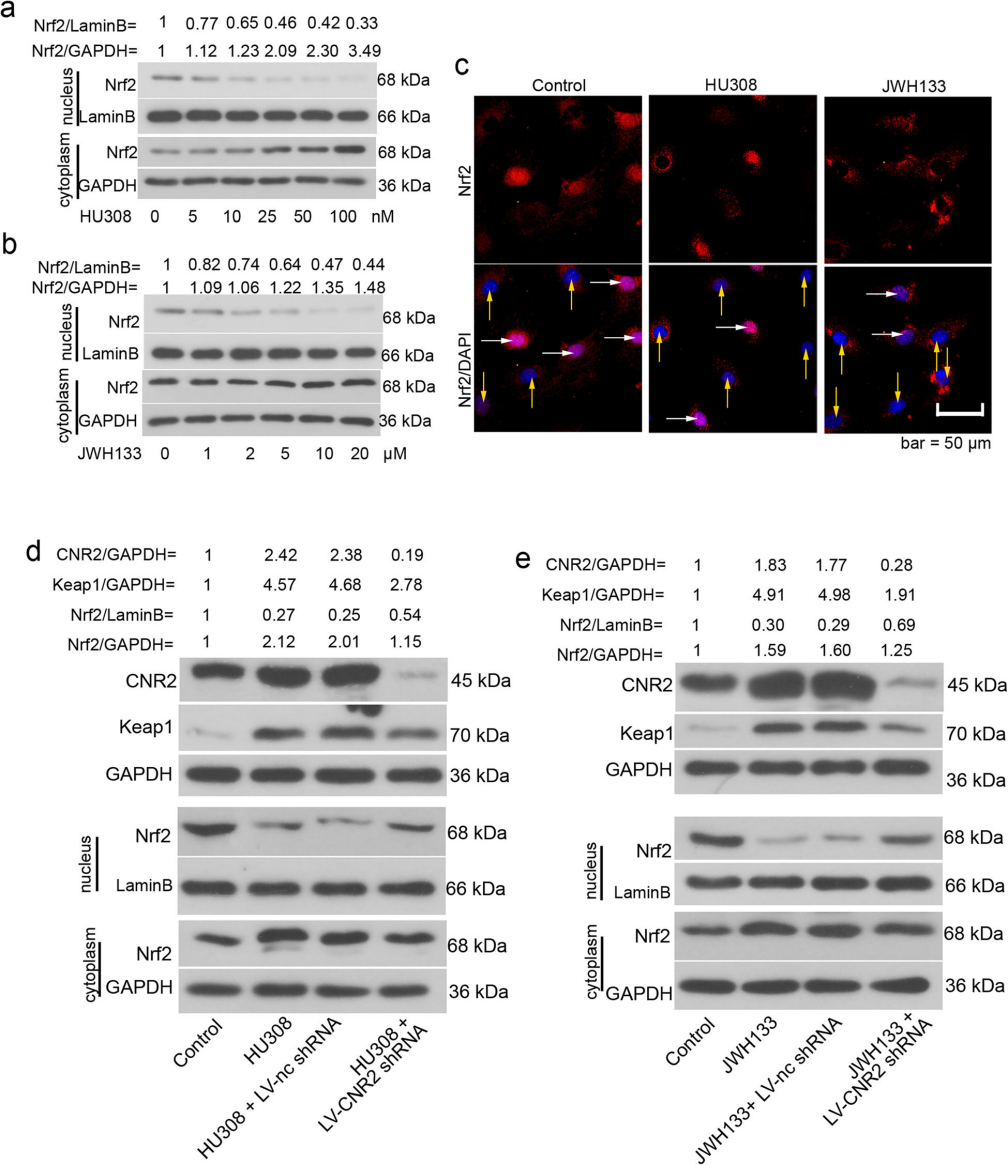
CNR2 agonists inhibit nuclear Nrf2 accumulation during hFOB 1.19 cell osteogenic differentiation. hFOB 1.19 cells were cultured at 34 °C until reaching confluence, and then transferred to 39 °C. Forty-eight hours later, HU308 (5, 10, 25, 50 or 100 nM) or JWH133 (1, 2, 5, 10 or 20 μM) was added into the cell medium. Twelve hours later, hFOB 1.19 cells were subjected to **a**-**b** western blotting analysis. **c** For immunofluorescence staining of Nrf2, hFOB 1.19 cells were treated with 50 nM HU308 or 10 μM JWH133 for 12 h. White arrows indicated cells positive to nuclear Nrf2, yellow arrows indicated cells negative to nuclear Nrf2. **d**-**e** hFOB 1.19 cells were infected with LV-CNR2 shRNA at 34 °C, and 24 h later, cells were transferred to 39 °C for differentiation. Forty-eight hours later, cells were further treated with 50 nM HU308 or 10 μM JWH133 for 12 h, and then harvested for western blotting analysis

hFOB 1.19 cells were cultured at 34 °C until reaching confluence, and then transferred to 39 °C. Forty-eight hours later, cells were exposed to varied concentrations of HU308 and JWH133 for another 12 h. Western blotting results illustrated that CNR2 agonists dose-dependently reduced nuclear Nrf2 accumulation in hFOB 1.19 cells (Fig. 4a-b). Immunofluorescence images validated western blotting results (Fig. 4c).

We determined the expression of CNR2 in hFOB 1.19 cells treated with HU308 or JWH133. Western blotting data indicated that CNR2 agonists induced CNR2 expression in hFOB 1.19 cells (Fig. 4d-e). Further, CNR2 shRNA successfully knocked CNR2 expression down in hFOB 1.19 cells (Fig. 4d-e). CNR2 silencing partly restored Nrf2 signals: nuclear Nrf2 increased and Keap1 expression decreased (Fig. 4d-e). Similar alterations were also observed in human BMMSCs (Additional file 1: Figure S1a-b). The above data suggest that CNR2 agonists trigger the deactivation of Nrf2 signaling pathway in hFOB 1.19 cells.

### CNR2 agonists promote p62 degradation, and induce release of Keap1 to retain Nrf2

**Fig. 5:**
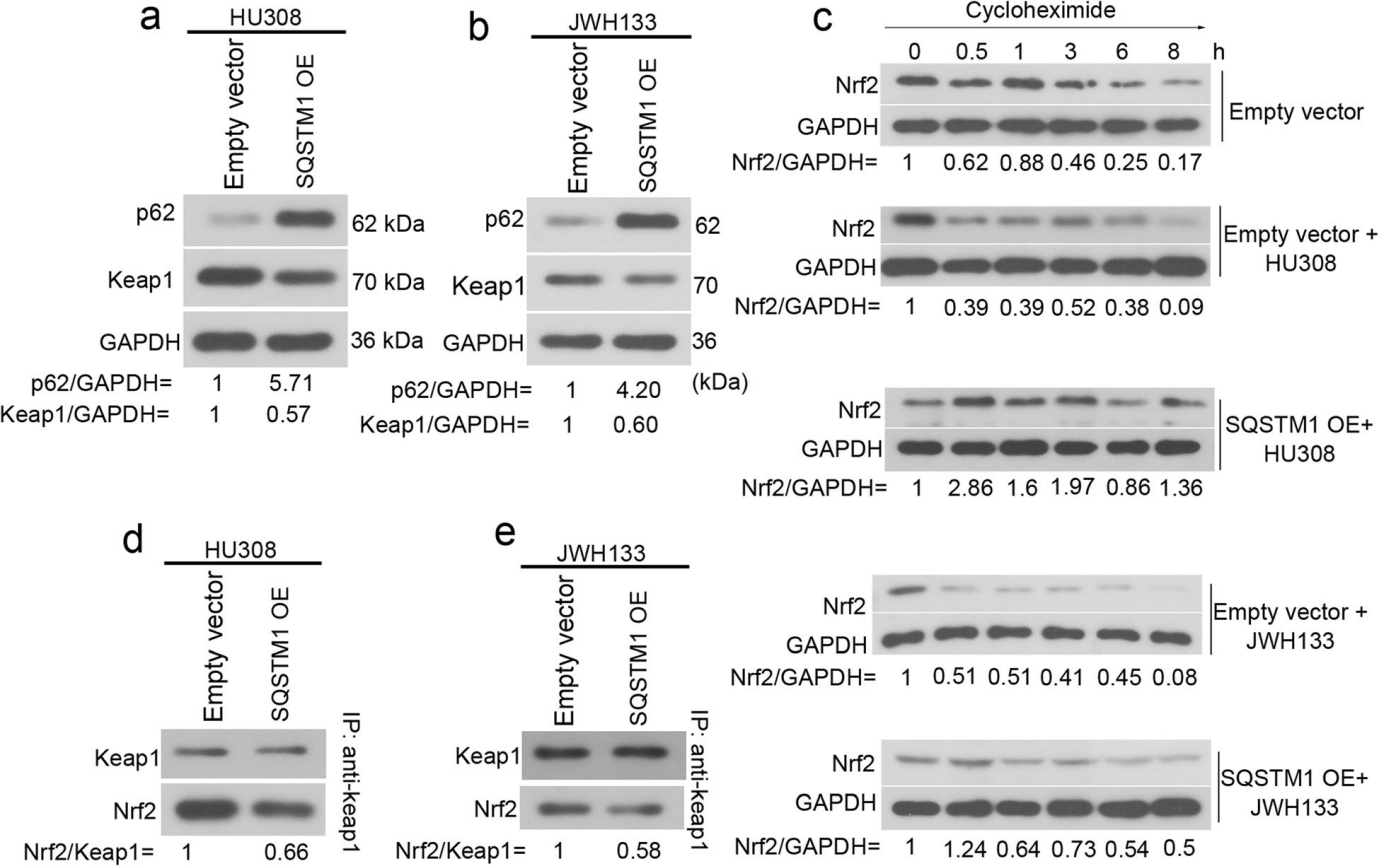
p62 stabilizes Nrf2 in hFOB 1.19 cells exposed to CNR2 agonists. hFOB 1.19 cells were cultured at 34 °C until reaching confluence and then transfected with empty vector or p62 overexpression vector (SQSTM1 OE). Then, culture medium was replaced, and cells were then cultured at 39 °C in presence of 50 nM HU308, 10 μM JWH133 for 48 h. **a**-**b** The protein levels of p62 and Keap1 were analyzed with western blotting. **c** Post a 48-h transfection, hFOB 1.19 cells were harvested at 0.5, 1, 3, 6, 8 h following treatment of 100 μg/mL cycloheximide. The expression levels of Nrf2 were determined with western blotting analysis. **d**-**e** For Co-IP assay, beads coated with anti-Keap1 was used to pull down intracellular Keap1, and the expression levels of Keap1 and Nrf2 were determined with western blotting

P62 is suggested to interact with Keap1-Nrf2 system [17]. In presence of HU308 or JWH133, the protein expression of p62 increased by 5.7- and 4.2-fold in hFOB 1.19 cells post the transfection of SQSTM1 (the gene encoding p62) OE plasmid (Fig. 5a-b). Keap1 expression decreased in response to p62 overexpression (Fig. 5a-b). To determine whether the degradation of Nrf2 was affected by CNR2 agonists and p62, the Nrf2 protein synthesis in hFOB 1.19 cells was first inhibited by 100 μg/ml cycloheximide. As indicated in Fig. 5c, degradation of Nrf2 protein started at 0.5 h (62%), and reduced to 17% at 8 h following cycloheximide treatment. Interestingly, CNR2 agonists significantly augmented Nrf2 degradation in hFOB 1.19 cells. In cells exposed to HU308, Nrf2 degraded to 39% at 0.5 h, and to 9% at 8 h (Fig. 5c). In cells exposed to JWH133, Nrf2 degraded to 51% at 0.5 h, and to 8% at 8 h (Fig. 5c). Moreover, CNR2 agonists-induced Nrf2 degradation was markedly attenuated in hFOB 1.19 cells overexpressing p62 (Fig. 5c). For Co-IP assay, beads coated with anti-Keap1 was used to pull down intracellular Keap1, and we found that less Nrf2 bound to Keap1 when p62 was overexpressed (Fig. 5d-e). These data together suggest that CNR2 agonists reduce p62 expression, thereby promoting Nrf2 degradation in hFOB 1.19 cells.

## Discussion

Our prior work has revealed the osteogenesis-promoting effects of CNR2 agonist in BMMSCs [11], and our current work further demonstrates an involvement of autophagy activation in such effects. We find that blockade of autophagy weakens the osteoinductive effects of HU308 and JWH133. CNR2 agonists-induced reduction in p62, an autophagy chaperone, contributes Nrf2 degradation in mature osteoblasts.

Under restrictive temperature (39 °C), hFOB 1.19 cells spontaneously differentiate towards mature osteoblasts [24]. CNR2 expression was previously demonstrated to increase with time in BMMSCs and MC3T3 E1 osteoblastic cells incubated in osteogenic medium [10], and here in differentiated hFOB 1.19 cells. These findings suggest that CNR2 elevation is associated with enhanced osteogenic differentiation. Interestingly, we noted that the basal autophagy of hFOB 1.19 cells was enhanced during the spontaneous differentiation period. Conversion of LC3I to LC3II was augmented, beclin1 expression was upregulated, and most importantly, p62 degraded significantly.

As an autophagy adaptor, p62 mediates the ubiquitin degradation of LC3 protein aggregates, and p62 itself is degraded by autophagy [25]. Thus, the successful degradation of p62 indicates an effective autophagic flux in differentiated hFOB 1.19 cells. On the basis of previous studies showing that CNR2 agonist HU308 augmented cellular autophagy [14, 15], we propose that CNR2 activation contributes to autophagy initiation in the differentiated osteoblasts. To valid this hypothesis, we treated hFOB 1.19 cells with increasing doses of CNR2 ligands HU308 and JWH133, and found that the autophagic flux was gradually enhanced. mTOR, a serine/threonine protein kinase, is a core component of mTOR complex 1 (mTORC1), the activation of which potently inhibits autophagy [26, 27]. Administration of mTOR inhibitor (rapamycin) or genetic ablation of mTOR has been reported to preserve the function of osteoblasts and osteocytes, and prevent bone loss [28, 29]. In addition to initiating autophagy, we also found that both CNR2 agonists suppressed mTOR signaling transduction. The inhibitory effects of HU308 on mTOR pathway in cardiomyocytes observed by Wu et al. [15] indirectly supported our current findings. Furthermore, CNR2 ligands’ effects on mTOR associated-autophagy were attenuated in CNR2-silenced cells. These data confirm that the activation of CNR2 signaling triggers autophagy in osteoblastic cells.

To explore how the autophagy activated by CNR2 agonists affects osteoblast differentiation, 3-MA was used to block autophagy in hFOB 1.19 cells. Interestingly, we found that CNR2 agonists-induced bone formation in vitro was attenuated when autophagy was blocked with 3-MA. These data suggest that autophagy induction is required for CNR2-medidated osteoblastic differentiation.

Bone formation is a dynamic process that is orchestrated by the balance between osteoblasts and osteoclasts [30]. Besides osteoblasts, the bone resorbing osteoclasts also generate endocannabinoids and express CNR2 [30]. While we demonstrate the osteogenesis-promoting effects of CNR2 agonists, others prove their osteoclastogenesis-promoting effects [8]. The opposite results revealing CNR2’s role in bone formation in osteoporotic animals [8, 10] may attribute to the enhanced differentiation of both bone formatting (osteoblasts) and absorbing (osteoclasts) cell lineages upon CNR2 activation. Furthermore, inhibition of autophagy with chloroquine suppressed osteoclast formation induced by receptor activator of nuclear factor kappa-B ligand (RANKL) [31]. This finding along with ours suggest that autophagy is involved not only in CNR2-regulated osteoblastic differentiation, but also in osteoclastic differentiation. Designing a cannabinoid-based anti-osteoporotic therapy should consider the dual role of CNR2 signaling in promoting osteoblast and osteoclast differentiation.

Oxidative stress acts a negative contributor to bone formation by inhibiting osteoblast differentiation, in which the Keap1-Nrf2 signaling plays an important role [32]. During the spontaneous osteogenic differentiation, we found that the basal expression of Nrf2 decreased in hFOB 1.19 cells. Adipose derived stem cells incubated in osteogenic medium also had reduced expression of Nrf2 [33]. This earlier study along with our present findings suggests that osteoblastic differentiation requires Nrf2 deactivation. Since CNR2 showed an opposite expression pattern to Nrf2 in hFOB 1.19 cells during differentiation, we assumed that CNR2 negatively regulated Nrf2 signaling transduction in osteoblasts. By further treating hFOB 1.19 cells with HU308 and JWH133, we noted that Nrf2 expression was downregulated, and that Keap1, a molecule known to mediate Nrf2 degradation [16, 17], was upregulated. Given to the negative role of over-activated Nrf2 signaling in regulating osteoblast differentiation [32], our results suggest that CNR2 activation-induced bone formation in vitro is associated with Nrf2 deactivation. Li and co-workers treated primarily cultured cardiac fibroblasts with another CNR2 agonist, AM1241, and found that, under oxygen and glucose deprivation condition, CNR2 activation further activated Nrf2 signaling transduction [34]. Such inconsistent result is from a total different cell lineage, which hardly disproves our current findings.

P62 stays in the center of autophagy and Keap1-Nrf2 system interaction [35]. Containing a KIR enables p62 to inactivate Keap1, thereby promoting Nrf2 activation [23]. As p62 degradation was induced by CNR2 ligands in hFOB 1.19 cells, additional experiments were performed to determine p62’s effects on Nrf2 expression in osteoblasts. Protein synthesis of hFOB 1.19 cells was blocked with cycloheximide, and the Nrf2 protein stabilization was determined. Interestingly, both HU308 and JWH133 accelerated Nrf2 degradation in hFOB 1.19 cells, which was prevented by forced overexpression of p62. These data reveal that CNR2 destabilizes Nrf2 by inducing p62 degradation in differentiated osteoblasts. As Nrf2 degradation is enhanced, it is predictable that more Keap1 binds to Nrf2 and leads to its ubiquitination mediated by ubiquitin E3 ligase complex. Nonetheless, to confirm this assumption, additional experiments detecting the ubiquitination of Nrf2 is needed.

hFOB 1.19 is an osteoblastic cell line established by transfection of temperature sensitive SV40 T-antigen. It has minimal chromosome abnormalities [24, 36], and thus is used as the in vitro model for studying the osteogenic differentiation. However, osteoblasts are actually derived from BMMSCs [37]. Hence, we further validated our major findings in human BMMSCs. Our data demonstrate an involvement of autophagy and Keap1-Nrf2 axis in CNR2-mediated osteoblastic differentiation only on the basis of data from the in vitro experiments. Using glucocorticoids- or ovariectomy-induced osteoporosic animal models will help to draw a more valid conclusion.

## Conclusion

Our work shows that CNR2 activation-induced osteoblastic differentiation in vitro is associated with autophagy induction and p62-mediated Nrf2 deactivation. These findings add novel insights into how CNR2-mediated cannabinoid signaling contributes to bone homeostasis.

## Supplementary information

**Additional file 1: Figure S1.** CNR2 agonists induce osteogenic differentiation and promote autophagy of human BMMSCs. To determine alterations in autophagy-associated molecules and Nrf2 signals, BMMSCs were infected with LV-CNR2 shRNA or control lentiviruses, and 24 h later, they were cultured in osteoinductive media for 48 h, and then treated with 50 nM HU308 or 10 μM JWH133 for 12 h. (a-b) The protein levels of indicated molecules were determined with western blotting analysis. BMMSCs were incubated in osteoinductive media in presence of 50 nM HU308, 10 μM JWH133 or 2 mM 3-MA. (c) The ALP activities and (d) the mRNA expression of osteopontin and osteocalcin of BMMSCs were determined after a 2-wk culture. (e) Cell mineralization was determined with Alizarin red staining after a 3-wk culture. Symbols ** and *** indicated a *p* value < 0.01 and < 0.001.

**Additional file 2: Figure S2.** 3-MA treatment hardly affects the vitality of hFOB 1.19 cells. hFOB 1.19 cells were cultured at 34 °C until reaching confluence, and then transferred to 39 °C. hFOB 1.19 cells were cultured at 34 °C until reaching confluence, and transferred to 39 °C. These cells were then treated with 2 mM 3-MA, 50 nM HU308 or 10 μM JWH133 for (a) 96 h or (b) 192 h, and their vitalities were determined with CCK8 assay.

## Data Availability

All data generated or analyzed during this study are included in this published article.

## Abbreviations

4EBP1: Eukaryotic Translation Initiation Factor 4E Binding Protein 1
ALP: Alkaline phosphatase
BGLAP/osteocalcin: Bone gamma-carboxyglutamate (gla) protein
CNR2: Cannabinoid receptor type 2
KEAP1: Kelch Like ECH Associated Protein 1
mTOR: Mammalian Target Of Rapamycin
Nrf2: Nuclear Factor, Erythroid 2 Like 2
P70S6K: Ribosomal Protein S6 Kinase 70 kDa Polypeptide 1
RNAi: RNA interference
SQSTM1/p62: Sequestosome 1
SSP1/osteopontin: Secreted Phosphoprotein 1
