# Supplementary figures and images for: Activation of cannabinoid receptor type 2-induced osteogenic differentiation involves autophagy induction and p62-mediated Nrf2 deactivation
Source: Cell Commun Signal. 2020 Jan 15;18:9. doi: 10.1186/s12964-020-0512-6 (PMC6964093; doi:10.1186/s12964-020-0512-6)

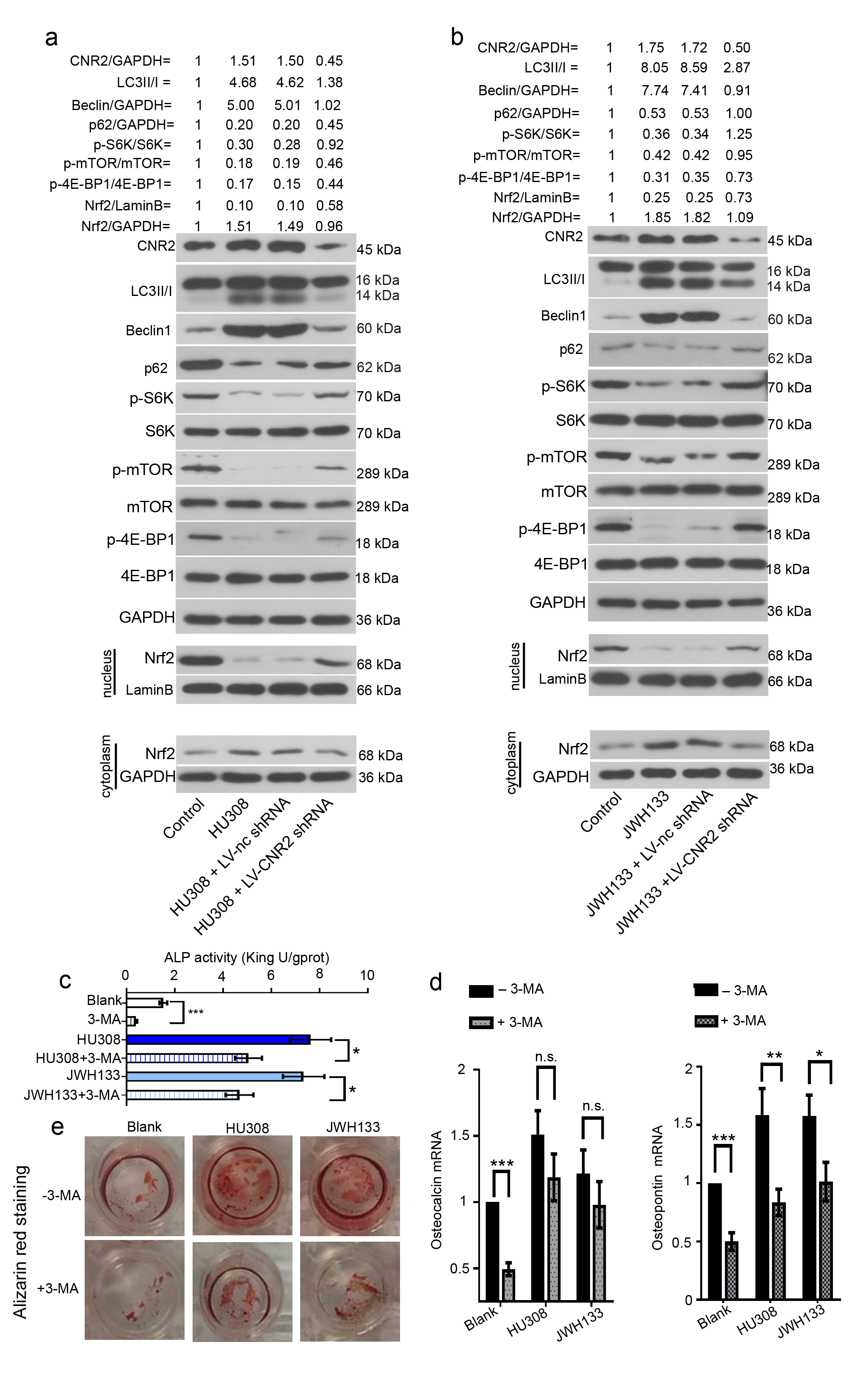

Supplement: Supplementary file 1 — Additional file 1: Figure S1. CNR2 agonists induce osteogenic differentiation and promote autophagy of human BMMSCs. To determine alterations in autophagy-associated molecules and Nrf2 signals, BMMSCs were infected with LV-CNR2 shRNA or control lentiviruses, and 24 h later, they were cultured in osteoinductive media for 48 h, and then treated with 50 nM HU308 or 10 μM JWH133 for 12 h. (a-b) The protein levels of indicated molecules were determined with western blotting analysis. BMMSCs were incubated in osteoinductive media in presence of 50 nM HU308, 10 μM JWH133 or 2 mM 3-MA. (c) The ALP activities and (d) the mRNA expression of osteopontin and osteocalcin of BMMSCs were determined after a 2-wk culture. (e) Cell mineralization was determined with Alizarin red staining after a 3-wk culture. Symbols ** and *** indicated a p value < 0.01 and < 0.001. [file 12964_2020_512_MOESM1_ESM.tif]

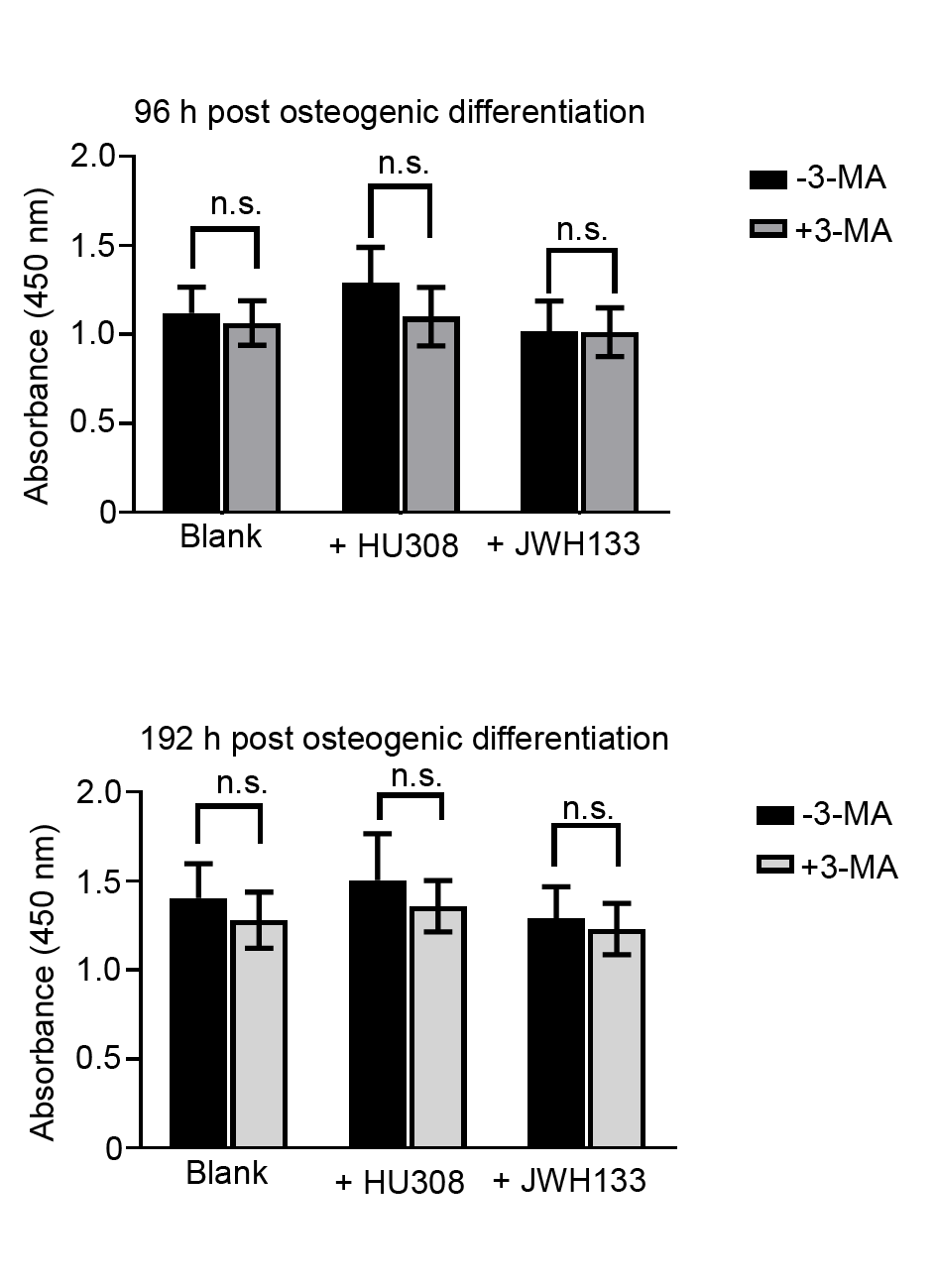

Supplement: Supplementary file 2 — Additional file 2: Figure S2. 3-MA treatment hardly affects the vitality of hFOB 1.19 cells. hFOB 1.19 cells were cultured at 34 °C until reaching confluence, and then transferred to 39 °C. hFOB 1.19 cells were cultured at 34 °C until reaching confluence, and transferred to 39 °C. These cells were then treated with 2 mM 3-MA, 50 nM HU308 or 10 μM JWH133 for (a) 96 h or (b) 192 h, and their vitalities were determined with CCK8 assay. [file 12964_2020_512_MOESM2_ESM.tif]
